# An Update on Emergent Nano-Therapeutic Strategies against Pediatric Brain Tumors

**DOI:** 10.3390/brainsci14020185

**Published:** 2024-02-18

**Authors:** Ammu V. V. V. Ravi Kiran, G. Kusuma Kumari, Praveen T. Krishnamurthy, Asha P. Johnson, Madhuchandra Kenchegowda, Riyaz Ali M. Osmani, Amr Selim Abu Lila, Afrasim Moin, H. V. Gangadharappa, Syed Mohd Danish Rizvi

**Affiliations:** 1 Department of Pharmacology, JSS College of Pharmacy, JSS Academy of Higher Education & Research, Rocklands, Ooty 643001, The Nilgiris, Tamil Nadu, India; devikiran006@gmail.com (A.V.V.V.R.K.); garikapatikusumakumari16@gmail.com (G.K.K.); praveentk@jssuni.edu.in (P.T.K.); 2 Department of Pharmaceutics, JSS College of Pharmacy, JSS Academy of Higher Education & Research, Mysuru 570015, Karnataka, India; ashapjohnson19@gmail.com (A.P.J.); madhuchandra152@gmail.com (M.K.); riyazosmani@gmail.com (R.A.M.O.); 3 Department of Pharmaceutics, College of Pharmacy, University of Ha’il, Ha’il 81442, Saudi Arabia; a.abulila@uoh.edu.sa (A.S.A.L.); a.moinuddin@uoh.edu.sa (A.M.)

**Keywords:** brain tumors, childhood cancers, pediatrics, nanoparticles, liposomes

## Abstract

Pediatric brain tumors are the major cause of pediatric cancer mortality. They comprise a diverse group of tumors with different developmental origins, genetic profiles, therapeutic options, and outcomes. Despite many technological advancements, the treatment of pediatric brain cancers has remained a challenge. Treatment options for pediatric brain cancers have been ineffective due to non-specificity, inability to cross the blood–brain barrier, and causing off-target side effects. In recent years, nanotechnological advancements in the medical field have proven to be effective in curing challenging cancers like brain tumors. Moreover, nanoparticles have emerged successfully, particularly in carrying larger payloads, as well as their stability, safety, and efficacy monitoring. In the present review, we will emphasize pediatric brain cancers, barriers to treating these cancers, and novel treatment options.

## 1. Introduction

Childhood/pediatric brain cancers are the second most common pediatric cancers, accounting for about one-fourth of all pediatric cancer cases [1]. The Industrial revolution and advancements in genetic screening and sequencing together ushered in new perspectives (both at the molecular and genetic levels) on these pediatric brain cancers. Mounting studies suggest that mutational burden is much lower in childhood brain cancers compared to adult brain cancers [1,2,3]. Furthermore, crucial targets in adult brain cancers cannot necessarily be exploited in childhood brain cancers due to their unique biology, which differs from adult cancers [1,4,5,6]. Over the past few decades, remarkable progress has been made in the treatment of childhood brain cancers, improving the patient survival rate by at least 5 years. Despite these improvements, many pediatric brain tumors are still incurable with high morbidity rates. Additionally, with the intensification of the therapy, the adverse effects of the chemo- and radiotherapies have become gradually apparent; for example, anthracyclines such as doxorubicin could cause cardiomyopathic problems [1,4].

In recent years, cancer nanomedicine has emerged as an important advancement in improving the therapeutic benefit [7,8]. Different nanoparticles, including organic, inorganic, or lipid-based nanoparticles, have been widely tested in delivering cancer theranostics. Further, these nanoparticles proved to be more advantageous than conventional methods due to their higher payload capacity, stability, and prolonged circulation time, thereby improving safety and efficacy [7,8]. In the present review, we will discuss the various pediatric brain cancers, barriers to treating these cancers, and novel treatment options. For selecting the recent relevant and informative research articles, a focused search using the keywords ‘brain tumors’, ‘childhood cancers’, ‘pediatrics’, ‘nanoparticles’, and ‘nanotheranostics’ was run using the databases such as Scopus, Web of Science, PubMed, ScienceDirect, Directory of Open Access Journals (DOAJ), etc. Articles showing more than 95% content and keyword match were included, and the rest were excluded.

## 2. Pediatric Brain Cancers: Targets and Mechanisms

Over the past few decades, cancer has been a long-lasting disease due to its heterogeneity [9,10]. In order to develop novel therapeutics, a thorough understanding of the underlying pathophysiological and molecular pathways is essential, especially in distinguishing childhood and adult cancers [11,12]. Unlike adult cancers, pediatric cancers are not triggered by lifestyle changes and are less inherited. Though the chance of risk development increases gradually with age, there are still a few exceptions. For instance, the occurrence of bone, brain, and blood cancers is greater in children than in adults. Further, the kinetic profile and therapeutic outcome differ in children than in adults (e.g., genitourinary pH, intestinal mobility, etc.) [11,13]. Additionally, pediatric tissues are immature and in the continuous growing phase, possessing greater metabolic rates and toxicity issues. Lastly, genetic variations in pediatric cancers, such as acute lymphocytic leukemia, Ewing sarcoma, etc., are greatly driven by fusion oncogenes due to chromosomal translocations. Unlike adult cancers, pediatric cancers possess a lower mutational rate, making their therapeutic targeting more challenging [13,14].

Considering the above-mentioned reasons, there is an imminent need to understand novel therapeutic targets and develop new therapeutic options. In this section, we will be discussing the various pediatric brain tumors and treatable options.

### 2.1. Medulloblastoma

Medulloblastoma (MB) is highly malignant and is the most common childhood brain cancer formed in the cerebellum [15]. MB is categorized into four molecular subgroups: Sonic hedgehog (SHH), WNT, group 3, and group 4 [15,16]. These subgroups are identified as powerful predictors of therapy outcomes. For instance, patients with WNT tumors have greater survival after therapy than group 3 tumors.

#### 2.1.1. WNT Subgroup

WNT-associated MB is most common in children over the age of three, with a 5-year survival rate, and is seldom metastatic. WNT-MB has no focal somatic copy number aberrations (SCNAs) and typically has chromosome 6 monosomy. Initially, it was recognized that people with Turcot syndrome, a genetic disorder caused by mutations in the adenomatous polyposis coli (APC) gene, a repressor of WNT signaling, had a higher incidence of MB [16]. Later, it was discovered that a subgroup of sporadic MBs had WNT pathway mutations, notably in CTNNB1 (encoding β-catenin). β-catenin enhances WNT target gene transcription by interacting with a number of chromatin modifiers such as histone acetyltransferases, SMARCA4, and CREBBP. Furthermore, whole-genome sequencing (WGS) has revealed that CTNNB1 mutations commonly coincide with missense variations in the DEAD-box RNA helicase DDX3X [17,18]. WNT-MB tumors are thought to form in the dorsal brainstem from progenitor cells in the lower rhombic lip.

Despite WNT-MB’s favorable prognosis, recent clinical trials have focused on lowering chemotherapy or radiation doses in the hopes of reducing off-target implications [16,17]. It is worth noting that WNT signaling has been proposed to play a role in WNT-MB’s exceptional response to standard therapy. Further studies revealed that these tumors release soluble WNT antagonists, which may disrupt the blood–brain barrier and sensitize tumors to chemotherapy.

#### 2.1.2. SHH Subgroup

Patients with SHH-activated MB were considered to have high-risk disease (survival rate of 50–75%), which is lower than WNT patients but higher than patients in group 3. SHH-MB, like WNT-MB, has a relatively even gender distribution. Unlike WNT-MB, most SHH-MB patients are newborns or adults; just a few youngsters have this tumor subtype. SHH-MB’s genome contains substantially more SCNAs than WNT-MB’s genome [13,17,18]. Initially, SHH signaling in MB was discovered in the context of Gorlin syndrome, a hereditary disorder embodied by basal cell carcinomas of the skin, craniofacial abnormalities, and an elevated prevalence of MB. Gorlin patients have germline mutations in PTCH1, a repressor of the SHH pathway. Germline mutations in the gene encoding Suppressor of Fused (SUFU) also predispose to SHH-MB [13,17]. Furthermore, spontaneous MBs in the SHH subgroup exhibit PTCH1 and SUFU loss-of-function mutations, Smoothened (SMO) and SHH activation mutations, GLI2, and MYCN amplifications. SHH pathway gene mutations are discovered in an age-dependent manner: All age groups have PTCH1 mutations; however, infants and adults are more likely to have SUFU mutations, infants are more likely to have SMO mutations, and children under the age of three are more likely to have MYCN and GLI2 amplifications. Mice with Gli2, Smo, Ptch1, and Sufu mutations are also susceptible to MB, indicating the function of these genes as tumorigenesis elicitors. A subset of SHH-MB patients, notably older adolescents and teenagers, have significant “chromothripsis” (chromosome shattering) [17]. Using WGS, Rausch et al. [19] discovered that these individuals typically had germline or somatic TP53 mutations, the former of which is related to Li-Fraumeni syndrome (LFS). Chromothripsis can cause SHH pathway gene amplification, such as GLI2 and MYCN, which increases SHH target gene expression and drives tumor development. For years, scientists have researched the genesis of SHH-MB tumors; however, most recent research indicates that these tumors are caused by granule neuron progenitors (GNPs). Small-molecule SHH pathway antagonists have made it possible to treat this subset of tumors in innovative ways. SMO inhibitors (SMOis), in particular GDC-0449 (vismodegib) and NVP-LDE225 (erismodegib), have been linked in clinical studies to strong (albeit frequently transitory) responses in MB patients [17]. SMO mutations may sometimes prevent long-term therapeutic benefits from occurring; in other circumstances, mutations in downstream components of the SHH pathway (for instance, MYCN or GLI2 amplifications) or in other pathways might render tumor cells resistant to these medications. The antifungal drug itraconazole and the cyclopamine derivative IPI-926 (saridegib) are two examples of second-site SMOis that have shown potential in preclinical research [20,21]. Arsenic trioxide, an inhibitor of downstream components of the SHH pathway, can accelerate GLI2 degradation [13].

#### 2.1.3. Group 3 Subgroup

Group 3-MBs account for about ~20–25% with very little prognosis and occur more in pediatrics than adults. At the time of diagnosis, most group 3-MB patients have the highest metastasis. Most group 3-MB originate in the midline in the proximity of the fourth ventricle of the brainstem [13,17]. The possibility of higher metastasis is due to this location, which facilitates access to the cerebrospinal fluid. Contrastingly, no germline mutations are known for the formation of group 3-MB [18]. The prominence of these tumors is majorly due to the amplification of the MYC oncogene, which fuses with the plasmacytoma variant translocation 1 (PVT1), which stabilizes the MYC proteins. Group 3-MB exhibits orthodenticle homeobox 2 (OTX2) amplification, which upregulates MYC expression, thereby promoting tumor formation. Further, genomic instability in group 3-MB is associated with the loss or gain of chromosomes. One of the key events in group 3-MB that happens in chromosome 17 is the simultaneous loss of 17p and gain of 17q chromosomes [18].

In order to identify novel therapeutics for group 3-MB, a high-throughput screening has been generated. The same study has revealed that gemcitabine and pemetrexed suppressed the group 3-MB in both mouse and human models. Despite improvement in survival having been observed in in vivo models, the involvement of tumor microenvironment has enhanced drug resistance in tumors. Another potential undruggable target in group 3-MBs is the overexpressed MYC gene. Treatment options for MYC can be achieved by bromodomain protein inhibitors such as JQ1, which arrest the G1 phase of the cell cycle, causing apoptosis [18].

#### 2.1.4. Group 4 Subgroup

Group 4-MBs account for more than one-third of all cases, with metastatic hallmark being the most common [13,18]. Unlike adult patients, infants and young adults with group 4-MBs have an intermediate survival rate. Similar to group 3-MBs, group 4-MBs originate adjacent to the fourth ventricle. Most of group-4 MBs possess chromosomal instability (esp. chromosome 17), causing the prevalence of SCNAs resulting from tetraploidization. Further, group 4-MBs possess < 10% mutations in KDM6A, ZMYM3, CTDNEP1, etc. An increase in SCNAs is reported to affect the NF-κB signaling, implying the potential therapeutic target [13]. Although the origin of group 4-MBs is unknown, gene signature has led to the glutamate-secreting neurons, suggesting from glutamatergic progenitors. Further studies revealed that the nuclear transitory zone (NTZ) is the main origin of group 4-MBs [22]. Due to the absence of a suitable animal model that mimics the group 4-MBs, proper therapeutic strategies against these tumors are limited. Few therapeutic options exist for treating group 4-MBs; for tumors that express MYCN and CDK6 amplification, bromodomain inhibitors and cyclin-dependent kinase (CDK) inhibitors along with MYC-destabilizing Aurora kinase A inhibitors may be preferred [23,24].

### 2.2. Gliomas/High-Grade Gliomas (HGGs)

Pediatric high-grade gliomas (HGGs) are the most common malignant brain tumors, majorly consisting of glioblastoma, astrocytoma, etc. [25]. World Health organization (WHO) has classified glioblastomas as grade IV due to their high proliferation, neovascularization, and necrosis [26,27]. Diffuse intrinsic pontine gliomas (DIPGs) are tumors that resemble gliomas histologically but possess diffusely metastatic growth inside the brainstem. Glioblastomas exhibit higher methylation of O6-methylguanine-DNA methyltransferase (MGMT), which results in impairment of DNA repair with the use of alkylating agents. To date, no therapeutic options have been available for improving survival for HGGs [26,27].

### 2.3. Neuroblastomas

Neuroblastomas are one of the major causes of death in pediatrics, accounting for about ~10–13% of all pediatric cancer cases [14,28]. Neuroblastomas originate from primordial neural crest cells, which form the adrenal medulla and sympathetic ganglia. Similar to glioblastomas, neuroblastomas also exhibit greater amplification of N-myc, which is associated with the expression of MRP and chromosome 1p deletion [28,29]. Overexpressed MRP on the neuroblastoma surface enhances the chemoresistance potential. Further, amplification of N-myc has downregulated the expression of CD44 receptors, which is a potential marker for aggressive tumor behavior. Additionally, low expression of Trk, a tyrosine kinase receptor, is associated with amplification of N-myc and even advanced stages of neuroblastomas. ALK amplification is another set of somatic mutations, accounting for ~14% of high-risk neuroblastomas [30]. Gain-of-function in ALK could drive the neuroblastoma but requires cooperation from MYCN amplification. Further, ALK upregulates the proto-oncogene RET and RET-driven sympathetic markers of the cholinergic lineage, which offer new therapeutic options, i.e., targeting both ALK and RET [30,31].

### 2.4. Ependymoma

Ependymoma (EPN) is one of the pediatric brain cancers which can occur in any part of the brain. The most popular originating location is the posterior fossa (cerebellum and brainstem), followed by supratentorial sites (cerebral hemispheres) and spinal cord [32,33]. The only option for treating ependymomas is surgery or radiation, as standard chemotherapy is ineffective. The molecular characteristics of EPNs are heterogeneous, mainly with dysregulation in growth factors such as epidermal growth factor receptor (EGFR), fibroblast growth factor receptor (FGFR), etc. EPNs originating from the posterior fossa (PF-EPN) are classified as PF-EPN-A and -B. PF-EPN-A is reported to be more deadly than PF-EPN-B [34]. Further, treatment with small molecule inhibitors, such as 3-deazaneplanocin A, causes degradation of PRC2 complex or with EZH2 (a GSK343 inhibitor), which competitively binds with S-adenosyl-L-methionine. EPNs originating from supratentorial (ST-EPNs) are reported to harbor fusion between RELA, NF-κB, and C11orf95 [35]. Further studies revealed that RELA fusion proteins alone could initiate the transformation of the neural stem cells [36]. Treatment options against ST-EPNs are mostly common chemotherapeutics such as temozolomide, vincristine, etc. [37] or HDAC inhibitors, including entinostat and vorinostat [38]. Ongoing research using preclinical models of ST-EPNs to evaluate potential druggable targets holds promise for developing treatment of tumors with RELA fusion proteins [32,34].

## 3. Nano-Based Approaches for Treating Pediatric Brain Cancers

**Figure 1:**
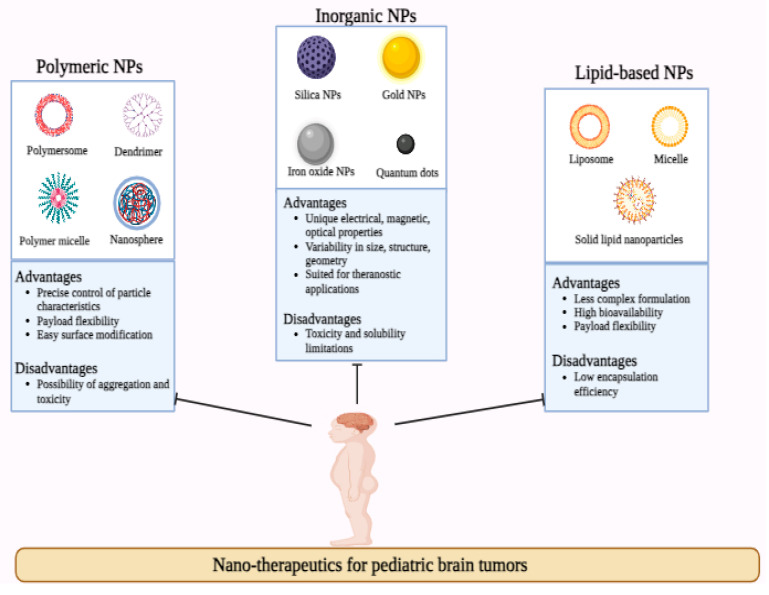
Schematic representation of nano-therapeutics for pediatric brain tumors.

Many research groups have emphasized utilizing nanotechnology to curb tumor progression, especially in pediatrics. In the present section, we will discuss nanotechnological advancements, especially for treating pediatric brain cancer. A schematic outline of diverse nanotherapeutic approached adopted for augmented pediatric brain cancer is depicted in Figure 1.

### 3.1. Nanotechnology and Blood–Brain Barrier

Due to its highly selective nature, the blood-brain-barrier (BBB) has become the first crucial barrier for many brain therapeutics and diagnostic entities. To develop a novel target-specific, an in-depth understanding of the physiology of BBB and overcoming strategies for nanoparticles is essential [39,40,41].

### 3.2. Physiology of BBB and Overcoming Strategies

BBB comprises highly specialized cells, which act as a protective barrier around the brain, especially in maintaining brain homeostasis. The cellular architecture of BBB mainly consists of brain capillary endothelial cells (BCECs), astrocytes, and pericytes [42]. Tight junctions between BCECs restrict the cellular diffusion of aqueous moieties. Further, in intact condition, BBB restricts the entry of ~99% of small drug molecules. BBB offers many transport mechanisms internally, i.e., via the transcellular lipophilic pathway, carrier-mediated transport (CMT), or receptor-mediated transport (RMT) [42,43]. However, the delivery of small molecules can be compromised by a large number of efflux pumps (such as adenosine triphosphate binding cassette transporters including multi-drug resistant protein (MRP) and p-glycoprotein (p-gp)), evade the foreign material into the bloodstream. Further, biological compounds, including inflammatory mediators (e.g., bradykinin, prostaglandin, vascular endothelial growth factors (VEGF)), signal receptors to increase BBB permeability [43].

Currently, drug delivery systems that are used in clinics are especially focused on local delivery. However, local delivery has limitations, including high rates of infection and excessive cerebrospinal fluid requirement. A non-invasive with direct delivery of therapeutic agents to the brain is the “intranasal route”, which is currently preferred but requires adjusting parameters such as dosage and positioning [42]. Transient opening of BBB can be achieved by biological (e.g., VEGF) or chemical stimuli (e.g., mannitol, oleic acid, cyclodextrins) but could have non-specific uptake, causing unwanted side effects. Therefore, an ideal approach to disrupting BBB is essential, one that would be controllable, reversible, specific, transient, and selective [43].

One of the best and most convenient approaches for drug delivery is intravenous administration with proper dosing. As discussed earlier, with the proper utilization of transporter proteins, specific receptors could be utilized for active targeting of nanoparticles-based drug deliveries. Further, disruption of BBB using either biological or chemical stimuli or nano-drug delivery systems, using either passive or active targeting, could achieve better BBB transport [41,42]. Moreover, most of the nano-drug delivery systems are lipophilic in nature, which is a crucial feature in bypassing the BBB.

**Figure 2:**
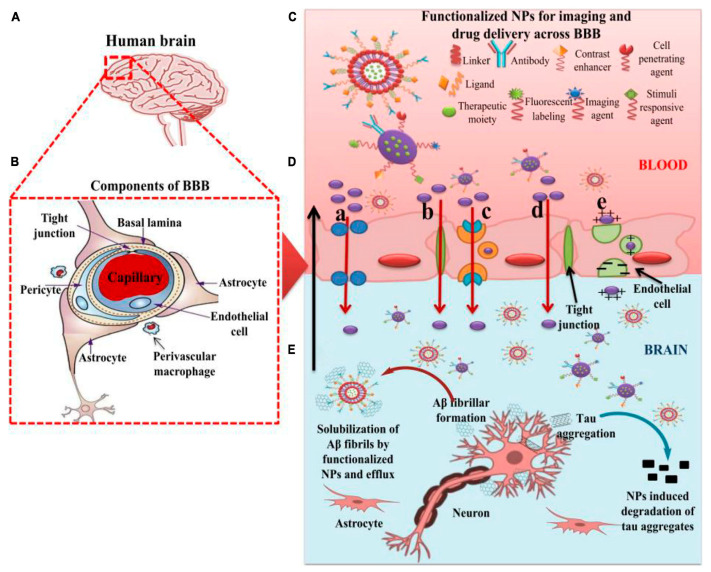
The role of nanoparticles in overcoming the BBB for efficient delivery of therapeutic moieties to treat AD. (**A**) Image of the human brain. (**B**) Components of the BBB. (**C**) Functionalized nanoparticles (NPs) for imaging and targeted drug delivery to the AD brain. (**D**) Different pathways of transport (a–e) across BBB are utilized by functionalized NPs. (a) Transport of NPs through cellular transport proteins; (b) transport of NPs through tight junctions; (c) transport of NPs via receptor-mediated transcytosis; (d) transport of NPs via transcellular pathway following diffusion, specifically adopted by gold NPs; and (e) transport of cationic NPs and liposomes via adsorption-mediated transcytosis. (**E**) Effect of functionalized NPs in treating AD via the degradation of tau aggregates and efflux of Aβ fibrils after getting solubilized by the NPs. AD: Alzheimer’s disease; NPs: nanoparticles; BBB: blood–brain barrier. Adapted with permission from [44].

Nanomedicines are small-sized nanocarriers that have been adopted to cure brain illnesses, including brain cancer and Alzheimer’s disease (AD). Functionalized nanoparticles are considered the most useful applicable approach to delivering these recommended drugs to the affected part of the brain. Nanomedicines have a set of unique properties that enable them to deliver anticancer drugs at target sites in the brain. Nanomedicines have the advantages of reduced dimensions and increased biocompatibility that facilitate the easy transport of therapeutic substances into the brain. Small-size nanomedicines can easily interact with the proteins and molecules on the cell surface as well as inside the cell. NP-functionalized nanomedicines have central core structures that ensure the encapsulation or conjugation of drugs and provide protection and prolonged circulation in the blood (Figure 2). Nanomedicines are also specialized to target cells or even an intracellular compartment and thus can deliver the drug at a predetermined dosage directly to the pathological site. Nanomedicines can minimize the dose and frequency and then improve patient compliance. Regardless of some clinical issues, nanomedicines have potential advantages of favorability to the brain, greater stability, biocompatibility and biodegradability, protection from enzymatic degradation, increased half-life, improved bioavailability, and controlled release over other conventional ways of drug delivery to the brain to cure AD (Figure 2) [44].

### 3.3. Nanoformulations Used for the Treatment of Medulloblastoma

Nanoparticle-based approaches are potential treatment options for pediatric medulloblastoma. NPs-based strategies mainly aim to improve the delivery of drugs by active or passive targeting and improve BBB crossing while reducing the side effects to surrounding healthy tissues.

Recently, herpes simplex virus type I thymidine kinase gene encoded plasmid loaded with poly (beta-amino ester) (PBAE) nanoparticles for gene therapy to medulloblastoma (MB) and atypical teratoid/rhabdoid tumors (AT/RT). The treatment with gene-encapsulated nanoparticles showed controlled apoptosis in transfected cells. In MB and AT/RT implanted mice, the gene therapy exhibited greater overall median survival [45]. An engineered biomimetic nanoparticle with dual targeting was designed to target the cancer stem-like cell population in sonic hedgehog medulloblastoma (SHH-MB). Treatment failure and poor outcomes are the significant struggles associated with the SHH-MB. High-density lipoprotein-mimetic nanoparticles (eHNPs) were used to cross BBB and load SHH inhibitors for the effective treatment of SHH-MB. Multi-component eHNPs were designed using microfluidic technology and are encapsulated with apolipoprotein A1, anti-CD15, and LDE225 (SHH inhibitor). eHNP-A1 improves the stability of the drug and has a therapeutic effect by SR-B1-mediated intracellular cholesterol depletion in tumor cells. These multifunctional nanoparticles exhibited promising effects in SHH-MB treatment and are applicable to other drugs that cannot cross BBB and have low bioavailability [46].

In a study, researchers developed a brain tumor model consisting of DAOY (MB cell lines) aggregates and cerebellum slices for evaluation of a poly(glycerol-adipate) (PGA) nanoparticle drug delivery system. PGA nanoparticles exhibited higher uptake than normal host cells. This novel tumor model suggests the effective evaluation of a drug delivery system between tumor cells and brain cells [47]. Kumar et al. [48] used Hedgehog inhibitor MDB5 and BRD4/PI3K dual inhibitor SF2523 to obtain the synergistic inhibition of medulloblastoma cell lines and to prevent resistance. They designed mPEG-b-PCC-g-DC copolymer-based NPs for effective loading of MDB5 and SF2523. NPs exhibit sustained release of loaded molecules. Targeted NPs were prepared by mixing COG-133-PEG-b-PBC and mPEG-b-PCC-g-DC copolymer and were found to be efficient in the reduction of tumors in orthotopic SHH-MB tumor-bearing NSG mice [48].

### 3.4. Nanoformulations Used for the Treatment of Glioma

Gliomas are the most common malignancy affecting the central nervous system. The primary treatment barrier for this disease is the difficulty of crossing the BBB by drug molecules. Nanoparticle-based approaches are familiar in overcoming these issues.

In a study, Temozolomide, an anti-glioma drug, was loaded on liposomes using proliposomes. The liposomes showed a slow release of temozolomide compared to the drug solution. The loading of temozolomide in liposomes improves the pharmacokinetic parameters compared to pure drug solution. Liposomes prolong the circulation time and improve the area under the curve (AUC). The biodistribution after IV injection revealed that the drug accumulation in the heart and lung is decreasing, and the concentration of the drug is increasing in the brain [49]. In another study, a targeted drug delivery platform was designed with PAMAM-PEG and transferrin for the encapsulation of temozolomide for the effective targeting of glioma stem cells. Glioma stem cells are responsible for the development of resistance. High cellular uptake and cytotoxicity were observed with transferrin-targeted temozolomide nanoparticles. The nanoparticle effectively crossed the BBB and delivered the drug specifically to the tumor. The PAMAM-PEG-trf nanoparticles induced potent cell apoptosis in drug-resistant glioma stem cells [50].

**Figure 3:**
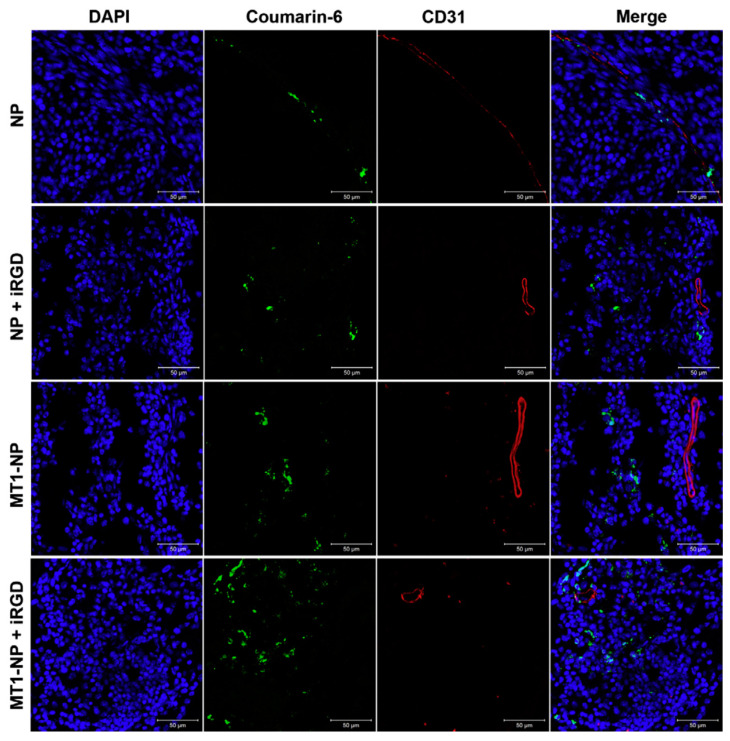
Observation of coumarin-6-labeled NP, NP co-administered with iRGD, MT1-NP, and MT1-NP co-administered with iRGD distribution in the brains of nude mice with intracranial C6 glioma, 3 h after intravenous administration. Analysis conducted on frozen sections using a confocal microscope revealed blood vessels marked with anti-CD31 (red), nuclei stained with DAPI (blue), and NPs depicted in green. Scale bars indicate 50 mm. Adapted with permission from reference [51].

Gu et al. [51] designed MT1-AF7p peptide-decorated paclitaxel-loaded PEG-PLA nanoparticles for glioma management. MT1-AF7p peptide has high binding to membrane type-1 matrix metalloproteinase (MT1-MMP) overexpressed on glioma cells. To improve the penetration of nanoparticles to glioma cells, the nanoparticles were co-administered with Tumor-homing and penetrating peptide iRGD. In C6 glioma cells, the peptide-decorated NPs showed significant cellular uptake via energy-dependent macropinocytosis and lipid raft-mediated endocytosis compared to non-peptide NPs. The nanoparticle’s extravasation across BBB and accumulation in glioma parenchyma was improved significantly in in vivo imaging and glioma distribution with MT1-AF7p functionalization and iRGD co-administration. Intracranial C6 glioma-bearing nude mice exhibited higher survival time with MT1-AF7p functionalization and iRGD co-administration (Figure 3) [51]. Bhunia et al. [52] have tailored a large amino acid transporter-1 (LAT1) conjugated nanometric liposomal carriers functionalized with amphiphile L-3,4-dihydroxyphenylalanine (L-DOPA) (Amphi-DOPA). Glioma-bearing mice showed higher uptake of NIR-dye labeled Amphi-DOPA to brain tissue. WP1066 labeled Amphi-DOPA enhanced the overall survivability of glioma-bearing C57BL/6J mice by 60% compared to the untreated group [52].

A red blood cell membrane-coated nanoparticle (RBCNP) with a neurotoxin-derived targeting ligand was designed for brain-targeted drug delivery. RBCNP can provide the biological function of natural cell membranes and desirable properties for drug delivery. The targeting moiety CDX peptide derived from candoxin has a high binding affinity to nicotinic acetylcholine receptors overexpressed on brain endothelial cells decorated on RBCNP. In vitro and in vivo results suggest that RBCNP-CDX has promising brain-targeting efficiency. In glioma mouse models, Dox-loaded RBCNP-CDX NPs showed superior therapeutic efficacy with less toxicity [53].

AS1411 aptamer functionalized poly (L-γ-glutamyl-glutamine)-paclitaxel (PGG-PTX) nanoconjugates were designed to achieve active targeting and optimized solubilization of paclitaxel. The tumor uptake of the nanoconjugate was mediated through nucleolin receptors, over-expressed in glioblastoma cells and neovascular endothelial cells. The in vivo fluorescence imaging and biodistribution studies suggest that the AS1411-PGG-PTX has higher tumor accumulation than PGG-PTX. In glioma-bearing mice, this nanoconjugate exhibited prolonged median survival time and most tumor cell apoptosis compared to PGG-PTX [54]. A dual-targeted liposome was designed for the co-delivery of doxorubicin (DOX) and vincristine (VCR) for glioma management. T7 (a ligand of transferrin receptors) and ^D^A7R (ligand of VEGFR 2) peptides were used to target glioma. The dual targeting strategy exhibited higher cellular uptake when compared to the single targeting strategy. The dual targeting and dual drug delivery showed the most favorable anti-glioma effect in vivo [55].

Nanocarrier-based immunotherapy has emerged as a promising approach for the treatment of various cancers, including glioblastoma. Gliomas are highly invasive. They usually infiltrate the normal tissues and make surgical removal of the tissue difficult. Immunotherapy in glioma can target and eliminate the infiltrating glioma cells to the neighboring tissues. Gliomas usually lead to an immunosuppressive environment and prevent normal immune cell reactions against cancer cells. Hence, immunotherapy is the best strategy to overcome the challenges and activate the immune system against tumor formation. Gliomas are known for their molecular and cellular heterogeneity. Immunotherapy can target specific antigens expressed on tumor cells, including tumor-associated antigens and neoantigens, addressing the diversity of cancer cells within the tumor [56,57].

Immunotherapy coupled with nanocarrier-based drug delivery presents a revolutionary approach to treating glioblastoma. Overcoming the BBB, nanocarriers enabled targeted delivery of immunotherapeutic agents, and enhanced drug bioavailability while minimizing systemic side effects are the advantages of nanocarrier-based immunotherapy in gliomas. The personalized nature of nanocarrier systems, tailored to individual tumor characteristics, promises a more effective and precise treatment. By encapsulating immunomodulatory agents, these carriers boost the immune response within the tumor microenvironment. Additionally, the ability to administer combination therapies and mitigate systemic toxicity underscores the potential of this innovative strategy in overcoming the challenges posed by glioblastoma, offering hope for improved patient outcomes. Checkpoint inhibitors, cytokines, and antigenic peptides are major immunomodulatory agents that can be delivered using nanocarriers to modulate the immune system’s response against glioblastoma [58,59].

Kuang et al. studied the effect of macrophage-directed immunotherapy with chemotherapy in orthotopic glioma. Doxorubicin and an immune checkpoint inhibitor (1-methyltryptophan, 1MT) were loaded on mesoporous silica nanoparticles modified with iRGD. The nanocarrier showed the ability to penetrate the BBB and accumulate drug molecules. The nanocarrier leads to the activation of cytotoxic CD8+ T lymphocytes and ions of CD4+ T cells in both GL261 cells cocultured with splenocytes in vitro and GL261-luc orthotopic tumors in vivo. The expression of antitumor cytokines was found to be upregulated, while protumor proteins were downregulated in the tumor tissues [60].

The Applications of Nanoparticles for medulloblastoma and glioma therapy are summarized in Table 1.

### 3.5. Nanoformulations Used for the Treatment of Neuroblastoma

Neuroblastoma (NB) is a complex pediatric tumor that originates from the neural crest and is the most common extracranial solid tumor in children, accounting for 15% of pediatric tumor-related deaths and 8–10% of all childhood malignancies. Most of the malignant cells are found in the adrenal medulla, but they can manifest as localized or metastatic tumors in the paraspinal ganglia, thorax, pelvis, and neck [67]. There are now several alternative therapeutic options for localized NB at different stages. Since most children are inoperable at the time of diagnosis due to metastases, even though complete resection of the primary NB is expected to greatly improve overall survival, the primary treatments in most cases still involve radiotherapy, chemotherapy, immunotherapy, differentiation-inducing therapy, and autologous hematopoietic stem cell transplantation [68]. Nano-based methods for treating pediatric neuroblastoma are an emerging area of interest and possible treatment plans in the upcoming years.

Treatment for neuroblastoma, a cancer of the nerve tissue that affects young children, is still challenging. When it comes to targeted therapy, imaging, and drug distribution for neuroblastoma, nano-based methods have distinct benefits. Solid lipid NPs, polymer micelles, nanoliposomes, nanocapsules, nanospheres, and nanomedicines are the primary forms of nanoparticles (NPs) [40]. Graphene oxide nanoribbons were developed by Mari et al. [69] to investigate their effects on human neuroblastoma cells. In one of the cell lines, they discovered that these nanoribbons stimulated autophagy and increased the synthesis of reactive oxygen species (ROS) within the first 48 h of exposure. Both cell lines observed a brief increase in ROS generation and autophagy at low doses; however, neither cell growth inhibition nor cell death was brought about by these effects [69]. Li et al. [70] studied zinc oxide nanoparticles (ZnO NPs) that were produced using Clausena lansium Peel aqueous extracts and zinc nitrate. These developed ZnO NPs were found to affect the regulation of autophagy and apoptotic proteins in SH-SY5Y neuroblastoma cells, leading to DNA damage, ROS generation, decreased cell stability, and viability. The utilization of N-acetyl-L-cysteine (NAC) was shown to mitigate ROS effects and prevent apoptosis, suggesting that ZnO NPs have the potential to induce cell death in neuroblastoma cells through the production of intracellular ROS. Kalashnikova et al. [71] developed and tested nanoceria and dextran-nanoceria formulations loaded with curcumin for treating childhood neuroblastoma. The formulations effectively killed neuroblastoma cells, particularly in MYCN-amplified cases, without damaging the healthy cells. This nanoparticle-induced oxidative stress stabilized HIF-1α and triggered the caspase-dependent apoptosis. These results offer a promising alternative to traditional drug therapies for aggressive cancers. Mohammadniaei et al. [72] developed a promising cell differentiation therapy using silver-coated bismuth selenide nanoparticles. The developed nanoparticles can be functionalized with a unique RNA structure to inhibit micro-RNA-17 and release retinoic acid, facilitating the transformation of cancer cells into neurons. This innovative research reports on the hydrophobicity challenges and offers a new method for drug delivery and real-time monitoring of the differentiation process, potentially advancing diagnostic and therapeutic agents. Zhang et al. [73] have demonstrated that Nab-paclitaxel exerted significant cytotoxicity against various pediatric solid tumor cell lines in vitro, with dose-dependent effects studied. In vivo studies on rhabdomyosarcoma and neuroblastoma xenograft models showed antitumor activity and increased survival in the metastatic model. Nab-paclitaxel induced tumor cell-cycle arrest and apoptosis, and its higher tumor/plasma drug ratio favored its efficacy compared to paclitaxel, even in paclitaxel-resistant relapsed tumors [73].

The applications of nanoparticles for neuroblastoma therapy are summarized in Table 2.

### 3.6. Nanoformulations Used for the Treatment of Retinoblastoma

Retinoblastoma is a disease in which malignant (cancer) cells form in the tissues of the retina. Retinoblastoma can expand to other parts of the body, such as the brain and spine [88]. Pediatric retinoblastoma (RB) is an uncommon and occasionally inherited malignancy. Because of alterations in the tumor-suppressor genes and the lack of a targeted, efficient, and cost-effective therapy, retinoblastoma is an uncommon form of cancer that is difficult to diagnose and treat. As such, there is a critical need for innovative treatments to address these issues [89]. External beam radiation, episcleral plaque radiation, cryotherapy, enucleation, and photocoagulation were conventional therapies for children with RB [90]. Ocular malignancies present unique problems, and improved penetration of the retinal pigment epithelium by monotherapies is required [91].

**Figure 4:**
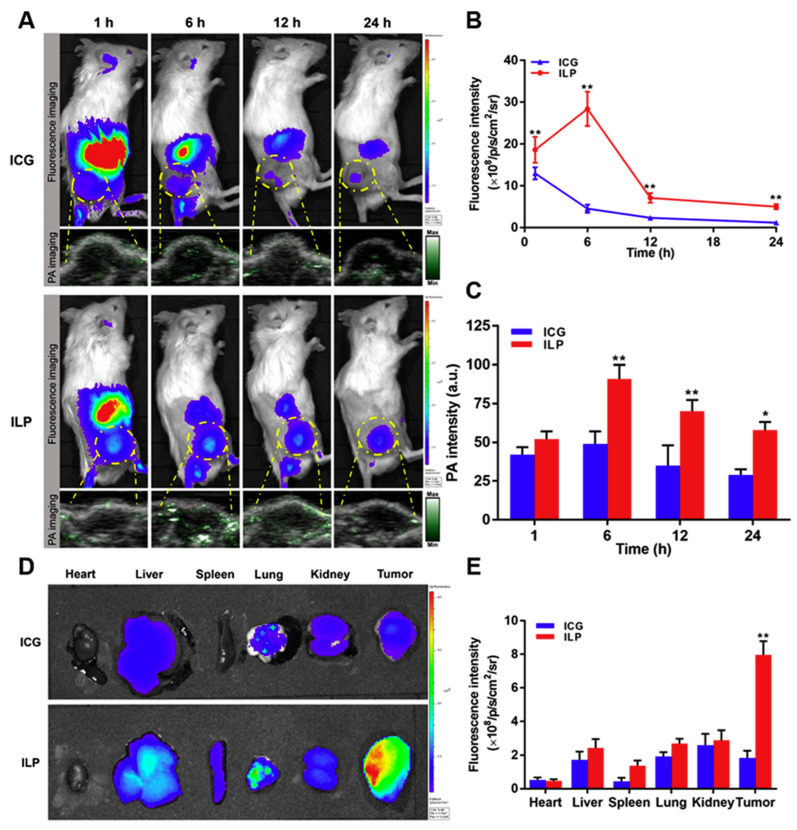
Assessing the fluorescence and photoacoustic imaging capabilities of ILP. (**A**) In vivo, observe the fluorescence and photoacoustic images of ICG and ILP at various time intervals. (**B**,**C**) Analyze the fluorescent intensity (**B**) and photoacoustic intensity (**C**) of tumor tissue quantitatively. (**D**) Examine ex vivo fluorescence images of ICG and ILP at 6 h. (**E**) Quantify the fluorescent intensity of different tissues at the 6 h mark. Herein *p* < 0.05 is flagged with one star (*) and *p* < 0.01 is flagged with two stars (**). Adapted with permission from reference [102].

Several nano-applications have been investigated recently to overcome these obstacles. The application of nanotechnologies in the detection and management of cancers and eye conditions has grown rapidly in recent years [92,93,94]. Among the most useful nanotechnology-based ocular delivery methods include nanoliposomes, polymeric nanoparticles (PNPs), nanocapsules, nanocages, nano-micelles, nano-dendrimers, and nanohydrogels, which offer several benefits over standard diagnostics and treatments [95,96,97]. Moradi et al. [98] assess the combined effects of gold nanoparticles (Au-NPs) and ultrasonic hyperthermia on Y79 cells. Cells were exposed to ultrasonic irradiation with or without 60 nm Au-NPs, and their viability was measured 48 h later. Results showed that hyperthermia alone reduced cell viability after 4 min, while in the presence of Au-NPs, this effect was observed after 4.5 min. Higher Au-NP concentrations increased cytotoxicity. This research concludes that the use of Au-NPs enhances the sensitivity of cells to hyperthermia induced by ultrasound [98]. A multifunctional nanoparticle system has been developed for the diagnosis and treatment of retinoblastoma. The nanoparticles, consisting of magnetic hollow mesoporous gold nanocages (AuNCs) loaded with muramyl dipeptide (MDP) and perfluoropentane (PFP), enable advanced imaging (photoacoustic, ultrasound, and magnetic resonance) for diagnosis and enhance low-intensity focused ultrasound (LIFU) therapy. These nanoparticles, when combined with LIFU, effectively target and treat RB tumors, leading to tumor cell death, while MDP activates dendritic cells (DCs) for improved immune response. The multifunctional nanoparticles offer potential for multimodal imaging-guided LIFU therapy and show promise for RB treatment with high safety [99]. Silver nanoparticles (AgNPs) are increasingly used in medical and commercial products due to their potent antibacterial properties. Rajanahalli et al. [100] have investigated the impact of AgNPs on mouse embryonic stem cells (mESCs). They revealed that AgNPs with different surface coatings altered cell morphology, induced cell cycle arrest at G1 and S phases, and reduced pluripotency marker Oct4A while promoting the expression of stress-related isoforms. The findings suggested that AgNPs’ toxicity is linked to excessive reactive oxygen species (ROS) production, with polysaccharide coating mitigating this effect [100]. Qu et al. [98,101] developed EpCAM-conjugated mesoporous silica nanoparticles (EpCMSN) to effectively deliver carboplatin (CRB) for the treatment of retinoblastoma (RB), a rare eye tumor. EpCMSN demonstrated enhanced cellular uptake and superior anticancer effects compared to free CRB, with a significantly lower IC_50_ value of 1.38 μg/mL. The specific receptor-mediated internalization of EpCMSN, targeting EpCAM receptors, suggests a promising approach for targeted treatment of RB and other ocular malignancies [101]. Photothermal therapy, with its minimal invasiveness and high specificity, addresses issues associated with traditional drug treatment for tumors. However, its limited tissue penetration hinders clinical application. Using a nano-platform comprising liposomes and indocyanine green (ICG) introduced a novel strategy for treating retinoblastoma by enhancing ICG stability and enabling imaging-guided photothermal therapy, making use of the eye’s transparency to infrared light. In this study, ICG-loaded liposome nanoparticles (ILP) were developed, offering targeted tumor treatment and improved imaging capabilities, holding promise for image-guided tumor phototherapy. Figure 4 depicts the fluorescence and photoacoustic imaging capabilities of ILP [102]. Cerium-doped titania nanoparticles (Ce-doped TiO_2_) were studied by Kartha et al. [103] using a cost-effective sol-gel method, and their enhanced photodynamic anticancer effects were evaluated on Y79 retinoblastoma cells. The study investigated the structural and optical properties of pure and Ce-doped TiO_2_, revealing cerium’s presence through X-ray diffraction and Raman spectra. Additionally, microscopy analysis showed that both TiO_2_ variants exhibited spherical shapes. The findings indicated that cerium doping in TiO_2_ enhances its photodynamic anticancer activity [103]. The applications of nanoparticles for retinoblastoma therapy are discussed in Table 3.

## 4. Clinical Trials

Pediatric brain tumors are some of the most devastating childhood diseases, with high mortality rates and significant long-term morbidity for survivors. Conventional treatment options like surgery, radiation, and chemotherapy often have severe side effects and limited efficacy. As mentioned in Table 4 (retrieved from https://clinicaltrials.gov/, accessed on 15 February 2024), emerging nanotherapeutic strategies offer a promising avenue for improving the treatment of pediatric brain tumors by overcoming these limitations. Nanoparticles, with their unique size and properties, can be designed to target tumor cells more effectively, deliver drugs with greater precision, and reduce systemic toxicity. Here is a brief overview of some ongoing clinical studies exploring nano-therapeutic strategies against pediatric brain tumors.

## 5. Conclusions

Pediatric brain tumors are considered the most frequent type of pediatric cancer, and they pose a tremendous therapeutic challenge owing to their tendency to infiltrate and disseminate to surrounding tissues, restricting the use of surgery as a feasible mono-therapeutic strategy. Furthermore, the difficulty in delivering medications to the brain tumor site in effective therapeutic concentrations while evading the blood–brain barrier (BBB) represents another challenge for cancer conquering. Consequently, nanomedicines have emerged as a promising therapeutic approach to circumvent the hurdles encountered with conventional therapy, along with improving the bioavailability of drug payloads. Nanotechnology-based delivery systems can effectively cross the BBB, and when decorated with receptors that are overexpressed both by BBB-building cells and cancer cells, they can discriminate cancer cells from surrounding healthy ones, thus directing the therapeutic agents towards malignant cells. However, various challenges must be carefully considered, including biocompatibility issues and clearance modulation. Nonetheless, various strategies have been implemented in recent years to overcome these drawbacks, and, along with the growing body of knowledge in the molecular genetics of brain tumors, the scientific community is unquestionably close to a major breakthrough in the development of efficient, safe, and low-cost nanosystems capable of imaging and treating brain cancers without inflicting remarkable damage to healthy tissue.

## Figures and Tables

**Table 1:** Applications of Nanoparticles for medulloblastoma and glioma therapy.

Carriers^ #^	Targeting Ligand^ #^	Targeting Receptor/Area^ #^	Therapeutic Molecule^ #^	Cell Line and/or Animal Model Used^ #^	Outcome/Key Findings^ #^	Ref.
Poly (beta-amino ester) (PBAE) nanoparticles	-	-	Herpes simplex virus type I thymidine kinase	AT/RT implanted mice	Greater median overall survival in mice implanted with AT/RT	[45]
High-density lipoprotein-mimetic nanoparticles	apolipoprotein A1 anti-CD15	SR-B1 CD15 antigen	LDE225	SHH MB cells	SR-B1-mediated intracellular cholesterol depletion in SHH MB cells.	[46]
mPEG-b-PCC-g-DC copolymer-based NPs	ApoE-targeting peptide COG-133	ApoE receptor	MDB5 SF2523	SHH-MB tumor-bearing NSG mice	Reduction in tumors in orthotopic SHH-MB tumor-bearing NSG mice	[48]
Liposomes	-	-	Temozolomide	Mice	Preferential accumulation in the brain	[49]
PAMAM-PEG- nanoparticles	Transferrin	Transferrin-1 receptors	Temozolomide	nude mouse intracranial xenograft models.	Anticancer activity against O6-methylguanine-DNA- methyltransferase gene promoter methylation.	[50]
PEG-PLA nanoparticles	MT1-AF7p peptide	Membrane type-1 matrix metalloproteinase	paclitaxel	C6 glioma-bearing nude mice	Enhanced survival time in intracranial C6 glioma-bearing nude mice	[51]
liposome	LAT 1	LAT-1 receptor	Amphi-DOPA	glioma-bearing C57BL/6J mice	Overall survivability increased by 60% in glioma-bearing C57BL/6J mice	[52]
RBC coated nanoparticle	CDX peptide	nAChRs	Doxorubicin	glioma bearing nude mice	High brain targeting, superior therapeutic activity with less toxicity	[53]
poly (L-γ-glutamyl-glutamine)- nanoconjugates	aptamer AS1411	Nucleolin	Paclitaxel	U87 MG cells and intracranial glioblastoma-bearing nude mice	Higher anti-glioma effect with enhanced median survival time	[54]
Liposome	T7 ^D^A7R	Transferrin receptors, VEGFR 2 receptors	Doxorubicin and vincristine	HUVEC cells, C6 Cells, glioma-bearing mice	High anti-glioma effect in in vivo studies	[55]
Chitosan-coated PLGA nanoparticles	-	Brain	Carmustine	U87 MG cell line Albino Wistar rats	Enhanced cytotoxicity in cell lines and AUC in brain	[61]
PG-SPIONs	Folic acid	Folate receptors	Lomustine	U87 MG cell line	Enhanced cellular uptake	[62]
CGT nanoparticles	CGT	integrins avβ3 and avβ5	-	Rat glioblastoma model	UTMD with CGT therapy improved the CGT delivery, prolonged tumor retention, apoptosis, and median survival period	[63]
Human serum albumin nanoparticles	Folic acid	Folate receptors	Erlotinib	U87MG and C6 cells rat glioblastoma model	Improved apoptosis and tumor reduction compared to pure drug	[64]
Liposomes	-	-	Doxorubicin and erlotinib	U87 MG cell lines	Improved apoptosis	[65]
poly (butyl cyanoacrylate) (PBCA) NPs	mAb	-	Carboplatin.	Rat glioblastoma model	Longer survival time	[66]

# ApoE—Apolipoprotein E, AT—Atypical Teratoid, CGT—Cilengitide, DOPA—Dihydroxyphenylalanine, HUVEC—Human Umbilical Vein Endothelial Cells, LAT 1—Large Amino Acid Transporter1, mAb—Mono clonal antibody, nAChRs—Nicotinic Acetylcholine Receptors, NSG—NOD Scid Gamma, PAMAM—Polyamidoamine, PBAE—Poly (Beta-Amino Ester), PEG-PLA—Poly (Ethylene Glycol)—Poly (Lactic Acid), PG-SPIONS—polyglycerol coated superparamagnetic iron oxide nanoparticles, RT—Rhabdoid Tumors, SHH-MB—Sonic Hedgehog Medulloblastoma, SR-B1—Scavenger Receptor Class B Type 1, UTMD—ultrasound-targeted microbubble destruction, VEGFR—Vascular Endothelial Growth Factor Receptor.

**Table 2:** Applications of nanoparticles for neuroblastoma therapy.

Carriers/Moieties^ #^	Therapeutic Molecule^ #^	In Vitro/In Vivo Models^ #^	Outcome/Key Findings^ #^	Ref.
Silica-PAMAM dendrimer Hybrid	Anthocyanins	Neuro-2A brain neuroblastoma from mouse and Vero (African green monkey kidney) normal cell lines	The Hybrid nanoparticles (134.8 nm) with +19.78 mV zeta potential showed effective cytotoxicity against Neuroblastoma (Neuro 2A) cells, with 87.9% inhibition due to anthocyanin release. This system appears to be primarily therapeutic in its current application. The placebo nanoparticles were non-toxic to the cells.	[74]
Liposomes	Pyrazolo[3,4-*d*]pyrimidines	Sprague Dawley rats	Liposomal encapsulation effectively overcame the poor water solubility of pyrazolo[3,4-*d*]pyrimidines; this study focuses on the therapeutic approach and makes them more suitable for clinical drug development.	[75]
PLA- and PLGA-based nanoparticles	Doxorubicin	Neuroblastoma cell line UKF-NB-3	These kinds of nanoparticles can enhance in vivo drug activity through the EPR effect and overcome transporter-mediated drug resistance. The primary focus of the study is on delivering doxorubicin as a therapeutic agent against neuroblastoma cells.	[76]
PLGA (Poly(lactic-co-glycolic acid) nanoparticles	Paclitaxel (taxol)	Human neuroblastoma cells (SH-SY5Y)	Paclitaxel-loaded PLGA nanoparticles exhibited cytotoxicity with cell viability below 50% at concentrations > 10 nM and induced genotoxic effects, suggesting their potential as a biocompatible carrier for neuroblastoma treatment and the developed system was used for a therapeutic approach.	[77]
Cyclodextrin-Fibrin gels (FBGs)	Doxorubicin (Dox)	Mouse orthotopic NB model (SHSY5YLuc+ cells implanted into the left adrenal gland	Increase in the therapeutic index of Dox when locally administered via FBGs loaded with oCD-NH2/Dox for neuroblastoma treatment. Overall, the research presented in the article focuses on improving the therapeutic efficacy of Dox delivery for neuroblastoma treatment, with possibilities for exploring theranostic applications in the future.	[78]
Biomimetic Core-Shell NNs	Therapeutic miRNA	Human neuroblastoma CHLA-255 cells and CHLA-255-luc tumor-bearing nonobese diabetic/severe combined immunodeficient (NOD/SCID) mice	NN/NKEXO cocktail for targeted neuroblastoma therapy, efficient miRNA delivery, dual tumor growth inhibition, and potential clinical application. Prepared systems are primarily focused on a therapeutic approach.	[79]
Soluplus or Chitosan Nanoparticles	Posidonia oceanica (POE)	SH-SY5Y human neuroblastoma cell line	NPs improved the aqueous solubility and stability of POE and enhanced its inhibitory effect on cancer cell migration, likely due to efficient encapsulation. Overall, the research presented in the article focuses on improving the therapeutic application of the nano-formulations.	[80]
Alginate-TiO2 TMZ Nanoparticles	Temozolomide (TMZ)	Human neuroblastoma cells SH-SY5Y	The developed system seems to focus primarily on a therapeutic approach, as these nanoparticles exhibit higher cytotoxicity against neuroblastoma cells, potentially impacting neuroblastoma treatment.	[81]
Solid lipid (Precirol^®^) and α_v_ integrins (ligand)	Etoposide and Cilengitide	HR-NB cell lines and MYCN-amplified cell lines	Combination therapy with cilengitide enhanced efficacy against high-risk neuroblastoma cells. This research offers theragnostic potential by targeting ECM-tumor cell interactions, inhibiting VN-integrin binding, modulating ECM stiffness, and employing nanoencapsulated chemotherapeutic agents to enhance the therapeutic index and overall effectiveness in high-risk neuroblastoma treatment.	[82]
Bacterial Membrane-coated Nanoparticle (BNP)	PC7A/CpG polyplex core with bacterial membrane	B78 melanoma tumors engrafted in syngeneic mice	BNP enhances immune recognition of tumor neoantigens post-radiation, improving dendritic cell uptake and cross-presentation, resulting in robust antitumor T-cell responses in mice with melanoma or neuroblastoma. The developed system seems to focus primarily on a therapeutic approach; therefore, the current application leans toward immunotherapy,	[83]
Synthetic High-Density Lipoprotein (HDL) Nanoparticles	4,19,27-tri acetyl withanolide A	Human NB cell lines SH-EP, SH-SY5Y, IMR32 and SK-N-As and tumor-bearing mice	Treatment reduced sphere formation, invasion, migration, and cancer stem cell markers in neuroblastoma cells. However, the targeting of SR-B1 and its potential for influencing CSC functions also suggest the potential for theranostic applications, where the nanoparticles could be used for both diagnosis and treatment.	[84]
Core-Shell MOF of Zinc	Titanocene Dichloride (TC) loaded Lactoferrin (Lf)	Neuroblastoma- IMR-32 cells and Wistar rats	ZIF-8 framework loaded with Lf-TC and 5-Fluorouracil exhibited potential for Neuroblastoma therapy, confirmed through in vitro cell studies and in vivo safety assessments in Wistar rats. The Lf-TC and 5FU-loaded ZIF-8 framework serves as nanoplatforms for tumor phototherapy, with the potential for transformation into a theranostic platform through additional imaging moiety modifications.	[85]
Graphene Quantum Dots (GQDs)	Anti-GD2 Antibody	NIH3T3 mouse fibroblast cell line and BE(2)-M17 human neuroblastoma cell line and nude mice	This study focuses on the theranostics potential of anti-GD2/GQDs and demonstrates the potential use of Anti-GD2/GQDs for targeting and imaging of neuroblastomas in vivo.	[86]
Nanocarriers coated-cationic liposomes functionalized with antibodies against GD2 receptor	miR-34a and let-7b	NB tumor cells, orthotopic xenografts, pseudometastatic models, athymic mice	Promising therapeutic efficacy of miR-34a and let-7b combined replacement, Support for clinical application as adjuvant therapy for high-risk NB patients.	[87]

^#^ NKEXOs: Natural killer cell-derived exosomes, NNs: core–shell nanoparticles, TiO_2_: Titanium dioxide, NB: Neuroblastoma, ECM: Extracellular matrix, CSC: Cancer Stem Cell, ZIF-8: Zeolitic Imidazolate Framework.

**Table 3:** Applications of nanoparticles for retinoblastoma therapy.

Carriers/Moieties^ #^	Therapeutic Molecule^ #^	In Vitro/In Vivo Models^ #^	Outcome/Key Findings^ #^	Ref.
AuNP-PEI-EpCAM Antibody (EpAb)	EpCAM-specific siRNA	Y79 retinoblastoma cells	Novel nanocarrier successfully delivered EpCAM-specific siRNA to retinoblastoma (RB) cells, leading to significant gene knockdown. The nanoparticles were well-tolerated by cells, and their conjugation with the EpCAM antibody enhanced internalization and therapeutic efficacy for RB. Gold nanoparticles also hold the potential for imaging in diagnosis.	[104]
Galactose-Chitosan Anchored Etoposide PLGA NPs (GC-ENP)	Etoposide (ETP)	Y-79 retinoblastoma cells	GC-ENP, with high entrapment efficiency and galactose targeting, demonstrates increased uptake in retinoblastoma cells (Y-79) and enhanced cytotoxicity, making it a promising drug delivery system for retinoblastoma treatment and enhancing the therapeutic application of the developed system.	[105]
PLGA Nanoparticles	Melphalan	Y79 cells	Surface modification improves efficacy in retinoblastoma cells, particularly with MPG-NPs. Prepared systems are primarily focused on a therapeutic approach, and this system enhances cell association, but some NPs remain on the cell surface rather than internalizing.	[106]
Nanospheres (NSs) of PGZ	Pioglitazone (PGZ)	Y-79 cell line and male pigs	The Polymeric nanoparticles effectively encapsulated PGZ, showing optimal characteristics with sustained drug release, good ocular tolerance, and significant in vivo anti-inflammatory potential, offering a promising approach for ocular inflammation treatment and suggesting a purely therapeutic approach.	[107]
CMD-TCs-NPs		Y79 retinoblastoma cells and Wistar albino rats	CMD-TCs-NPs show smaller size, positive zeta potential, and higher affinity for retinoblastoma tumors in rat eyes when administered intravitreally, while CMD-TMC-NPs remained in the vitreous and did not reach the retina. These findings suggest CMD-TCs-NPs’ potential for more effective drug delivery in retinoblastoma treatment.	[108]
Thiolated Chitosan Nanoparticles (TPH-TCs-NPs)	Topotecan (TPH)	Human retinoblastoma cells (Y79), xenograft-rat-model of retinoblastoma	TPH-TCs-NPs enhanced drug loading, improved control over drug release, and increased treatment efficacy for retinoblastoma. Thiolated chitosan demonstrates improved interaction with cell membranes, leading to higher cellular uptake of the drug. Therefore, this study primarily focuses on the therapeutic approach.	[109]
siRNA-loaded switchable LNP	Survivin siRNA	Y79 retinoblastoma cells and primary human RB cells.	Sequential siRNA survivin followed by chemotherapy sensitizes cancer cells to carboplatin and melphalan, showing promise in treating retinoblastoma (RB) without affecting healthy cells. The study suggests careful drug screening to find synergy with survivin for future in vivo testing.	[110]
Lipid Nanoparticles (LNP)	Melphalan and miR-181a	Y79 retinoblastoma cells and Sprague Dawley rats	Co-delivery of melphalan and miR-181a using 171 nm switchable LNP with high encapsulation efficiencies enhanced therapeutic efficiency, reducing the expression of anti-proliferative and anti-apoptotic genes while increasing pro-apoptotic gene expression.	[111]
Lactoferrin nanoparticles (Lf-Nps)	Carboplatin (CPT) and Etoposide (ETP)	Retinoblastoma (Rb) Y79 cells	The Nanoformulations of Lf-CPT and Lf-ETP enhance drug uptake, intracellular retention, and cytotoxicity, particularly in Rb Y79 CSCs, offering the potential for improved targeted therapy and therapeutic efficacy and better clinical outcomes by overcoming chemoresistance in cancer stem cells (CSCs).	[112]
Apo-nano-carbo and Lacto-nano-carbo nanoparticles	Carboplatin	Human retinoblastoma cell line Y79	These nanoparticles demonstrated pH-dependent drug release and receptor-mediated endocytosis for targeted delivery, resulting in greater intracellular uptake and anti-proliferative activity (IC_50_ = 4.31 μg ml^−1^ and 4.16 μg ml^−1^, respectively) compared to soluble carboplatin (IC50 = 13.498 μg ml^−1^).	[113]
Polymethylmethacrylate nanoparticles	Carboplatin	Sprague Dawley rats	Intra-vitreal carboplatin concentrations were significantly higher with novel carboplatin-loaded polymethylmethacrylate nanoparticles (NPC) compared to the commercially available carboplatin (CAC), indicating enhanced trans-scleral permeability for potential use in treating advanced retinoblastoma. Therefore, this study primarily focuses on the therapeutic approach.	[114]
Folic Acid-Conjugated Polymeric Micelles	Curcumin-Difluorinated (CDF)	Retinoblastoma cell lines (Y-79 and WERI-RB1)	The Folic acid-conjugated micelles loaded with CDF increased CDF solubility and showed significant anticancer activity on retinoblastoma cell lines (Y-79 and WERI-RB). This formulation holds promise as an alternative approach to retinoblastoma therapies; therefore, the study focuses on the therapeutic potential of the developed system.	[115]
EpCAM antibody-functionalized PLGA NPs.	Paclitaxel	Y79 retinoblastoma cells	EpCAM antibody-functionalized biodegradable NPs show potential for tumor-selective drug delivery and overcoming drug resistance in retinoblastoma treatment. Therefore, this study primarily focuses on the therapeutic approach.	[116]
Hybrid Lipid Polymer Nanoparticles	Beta-lapachone (β-Lap)	Retinoblastoma cells	This study focuses on a combined chemo- and photodynamic therapy (PDT) approach, aiming to synergistically treat retinoblastoma with both β-Lap and m-THPC encapsulated in LNPs.	[117]
Mesoporous silica nanoparticles	anti-MRC2 and/or anti-CD209	Human retinoblastoma cancer cells (Y-79 and WERI-Rb1)	Identifies elevated expression of two receptors, MRC2 and CD209, in retinoblastoma, leading to the creation of mesoporous silica nanoparticles (MSN) equipped with anti-MRC2 and/or anti-CD209 antibodies for targeted PDT and imaging.	[118]

# AuNP-PEI-EpCAM Antibody: Gold nanoparticles-polyethyleneimine-Epithelial cell adhesion molecule monoclonal antibody conjugated, CMD-TCs-NPs thiolated and methylated chitosan-carboxymethyl dextran nanoparticles, siLNP: siRNA-loaded switchable lipid nanoparticles, Apo-nano-carbo: carboplatin loaded apotranferrin and Lacto-nano-carbo: lactoferrin loaded nanoparticles, PLGA NPs: Poly(lactic-co-glycolic acid) nanoparticles, PDT: Photodynamic therapy.

**Table 4:** Clinical trials using nano-therapeutics in pediatric brain tumors.

Carriers/Nanoparticles^ #^	Condition^ #^	Therapeutic Agent^ #^	Phase^ #^	Status^ #^	NCT Code
Liposomes	Glioblastoma	C225-ILs-dox	Phase 1	Completed	NCT03603379
Gold Nanoparticle	Recurrent Glioblastoma	NU-0129 IV	Early Phase 1	Completed	NCT03020017
Ultra-small iron oxide particle	Brain Neoplasms	Combidex as MRI contrast agent	Phase 2	Terminated	NCT00659334
Small iron particles	Childhood Brain Neoplasm	DSC-MRI with ferumoxytol	Early Phase 1	Completed	NCT00978562
Nanoparticle Formulation MTX110	Diffuse Intrinsic Pontine Glioma	Panobinostat	Phase 1 Phase 2	Completed	NCT03566199
Liposomes	Brain tumor	Doxorubicin	Phase 1	Completed	NCT00019630
Liposomes	Brain and Central Nervous System Tumors	Cytarabine	Phase 1	Unknown status	NCT00003073
MTX110 and gadolinium	Diffuse Intrinsic Pontine Glioma	Infusate	Phase 1	Completed	NCT04264143
Liposomes	Neuroblastoma	Doxorubicin	Phase 1	Terminated	NCT02536183
Liposome	Neuroblastoma	Doxorubicin	Phase 1	Withdrawn	NCT02557854
Liposome	Neuroblastoma	Irinotecan Sucrosofate	Phase 1	Recruiting	NCT02013336

# C225-ILs-dox: Doxorubicin-loaded Anti-EGFR-immunoliposomes.

## Data Availability

Data are contained within the article.
